# Data on the impact of increasing the W amount on the mass density and compressive properties of Ni–W alloys processed by spark plasma sintering

**DOI:** 10.1016/j.dib.2016.04.011

**Published:** 2016-04-11

**Authors:** T. Sadat, A. Hocini, L. Lilensten, D. Faurie, D. Tingaud, G. Dirras

**Affiliations:** a Université Paris 13, Sorbonne-Paris-Cité, LSPM (UPR 3407), CNRS, 99 Avenue Jean-Baptiste Clément, 93430 Villetaneuse, France; b Université Paris Est, ICMPE (UMR 7182), CNRS, UPEC, 94320 Thiais, France

**Keywords:** Ni–W alloys, Spark plasma sintering, Compression tests, Density

## Abstract

Bulk Ni–W alloys having composite-like microstructures are processed by spark plasma sintering (SPS) route of Ni and W powder blends as reported in a recent study of Sadat et al. (2016) (DOI of original article: doi:10.1016/j.matdes.2015.10.083) [1]. The present dataset deals with determination of mass density and evaluation of room temperature compressive mechanical properties as function of the amount of W (%wt. basis). The presented data concern: (i) measurement of the mass of each investigated Ni–W alloy which is subsequently used to compute the mass density of the alloy and (ii) the raw (stress (MPa) and strain ($\frac{\Delta L}{L_{0}}$)) data, which can be subsequently used for stress/ strain plots.

**Specifications table**

**Table:** 

Subject area	*Materials Science and Engineering*
More specific subject area	*Material Characterization / Mechanical properties*
Type of data	*Table and graphs (excel spreadsheets)*
How data was acquired	*Following techniques were used for acquiring data: Room temperature quasi-static compression testing, mass measurements*
Data format	*Raw, filtered*
Experimental factors	*Room temperature quasi-static Compression results.*
*Mass and density measurements*
Experimental features	*Brief experimental description*
Data source location	*LSPM - University Paris 13, Villetaneuse, France (compression tests and weight measurements).*
Data accessibility	*Data are included in this article*

**Value of the data**

• The present dataset shows the impact of the addition of W on the evolution of the mass density and on the room temperature compression properties, particularly the yield strength.
• The presented data may serve as a basis for designing and tailoring on demand Ni–W alloys (or more generally composite-like microstructure) with a given combination of properties.
• The data can also serve as a benchmark for other researchers interested in comparing and/or use of these values, or develop the best alloy for a given application.
• Engineering stress versus strain or true-stress versus true-strain data can be plotted (from raw data) and mechanical properties under quasi-static compression at room temperature can be evaluated.

## 1 Data

Weight measurements of each Ni-W alloys inside and outside distilled water are carried out. The obtained values are used to compute the alloy mass density.

Raw stress-displacement data of the processed materials tested under quasi-static compression at room temperature are also presented.

## 2 Experimental design, materials and methods

Ni–W alloys and pure (unalloyed) Ni sample are obtained by spark plasma sintering (SPS) process of powder blends (with controlled increasing amount of W) and unalloyed Ni powder, respectively [1].

Compression tests are carried out at room temperature at a strain rate of 10^−3^ s^−1^. Prismatic specimens having a surface to height ratio of about 1.8 mm are used. The compression tests are conducted using a 100 kN MTS testing machine (model 20/MH). The strain is deduced from the crosshead displacement and corrected by the stiffness of the machine. A thin layer of teflon is added between the sample and the compression platens to reduce friction and any shearing effects.

The true stress - true strain values are obtained from the engineering stress-strain values by considering the equations below:

$$\sigma_{\mathrm{true}}=\sigma \times (1-\left|Ɛ\right|)$$

$${Ɛ}_{\mathrm{true}}=-\log(1-\left|Ɛ\right|)$$

where:

• *σ* is the engineering stress of the sample (MPa).
• Ɛ is the engineering strain of the sample.
  The mass density is determined by the Archimedes method at room temperature using a Mettler Toledo XP105 scale. The following formula is used to compute the mass density:
  $$\rho =\frac{A}{A-B}\left(\rho_{0}-\rho_{L}\right)+\rho_{L}$$
  where:
• ρ is the density of the volumetric mass density of the sample (g/cm^3^)
• *A* is the mass of the sample in the air (g).
• *B* is the mass of the sample in the liquid (distilled water) (g)
• $\rho_{0}$ is the volumetric mass density of the fluid (g/cm^3^)
• $\rho_{L}$ is the volumetric mass density of the air (g/cm^3^)

**Fig. 1:**
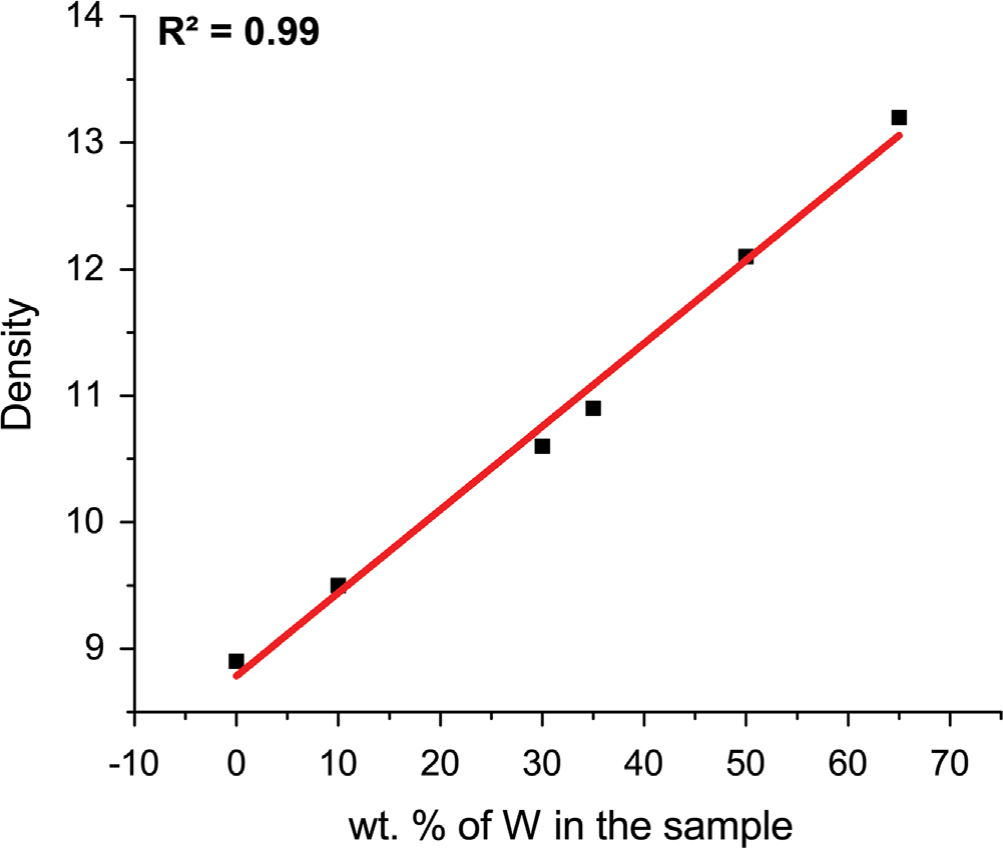
Density *vs* the initial W amount (wt.% basis).

Table 1: summarizes the obtained data. The mass density increases linearly with the amount of W (Fig. 1) with a coefficient of determination of 0.99.

**Fig. 2:**
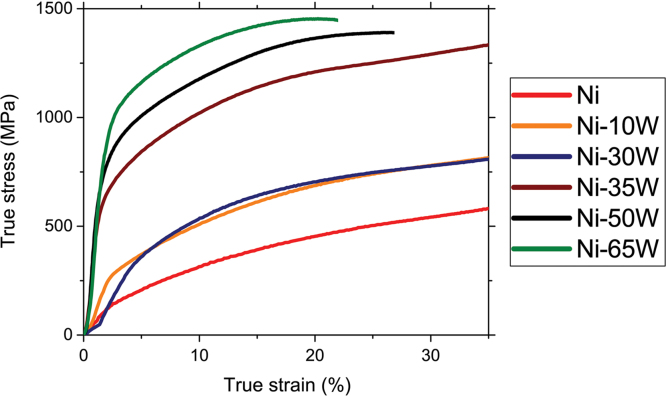
True stress *vs* true strain plots after room temperature compression tests of the unalloyed Ni and the Ni-10W, Ni-30W, Ni-35W, Ni-50W and Ni-65W alloys.

**Fig. 3:**
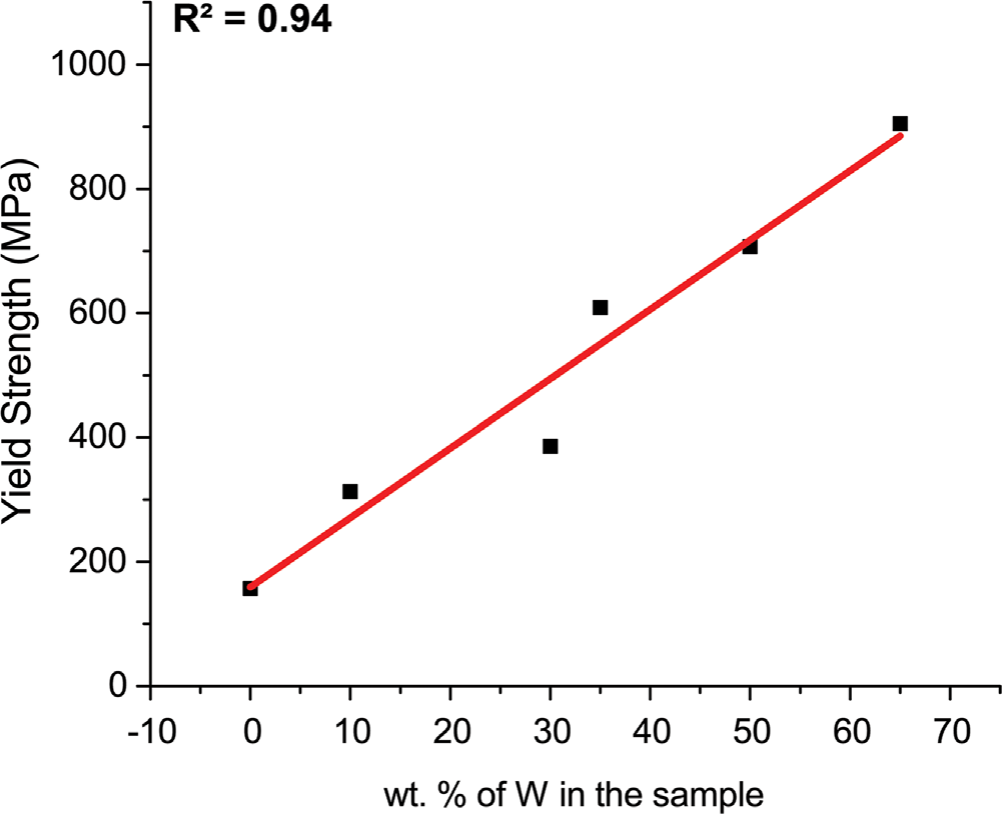
Yield strength vs the initial amount (wt.% basis).

As an example of the use of the supplied data presented here, the true stress versus true strain plots for the different alloys are presented in Fig.2. The obtained plots clearly illustrate the impact of W addition: the yield strength and the strength increase with increasing the amount of W. Similar behavior has been reported elsewhere [2]. Moreover, Fig. 2 shows that samples containing W amount higher than 30 wt.% exhibits the higher apparent Young modulus. It is also interesting to note, as shown in Fig. 3, that the yield strength linearly increases with increasing the amount of W.

The linear behaviors can be used to predict microstructures with a given combination of alloys’ properties such as mass density and yield strength.

## Figures and Tables

**Table 1:** Mass density, yield strength and true compression strength.

Sample name	W content (wt. %)	Density	Yield strength (MPa)	True strength (MPa)
Ni	0	8.9	134	701
Ni-10W	10	9.5	249	905
Ni-30W	30	10.6	334	895
Ni-35W	35	10.9	618	1451
Ni-50W	50	12.1	807	1391
Ni-65W	65	13.2	951	1453
