# Supplementary material for: Data on the impact of increasing the W amount on the mass density and compressive properties of Ni–W alloys processed by spark plasma sintering
Source: Data Brief. 2016 Apr 11;7:1405–8. doi: 10.1016/j.dib.2016.04.011 (PMC4845081; doi:10.1016/j.dib.2016.04.011)
Supplement: Supplementary file 3 — Supplementary material [file mmc3.pdf]

Conflicts of Interest Statement

Manuscript title: Data on the impact of increasing the W amount on the mass density and compressive properties of Ni-W alloys processed by spark plasma sintering.

The authors whose names are listed immediately below certify that they have NO affiliations with or involvement in any organization or entity with any financial interest (such as honoraria; educational grants; participation in speakers' bureaus; membership, employment, consultancies, stock ownership, or other equity interest; and expert testimony or patent-licensing arrangements), or non-financial interest (such as personal or professional relationships, affiliations, knowledge or beliefs) in the subject matter or materials discussed in this manuscript.

Author names:

Tarik SADAT
Azziz Hocini
Lola LILENSTEN
Damien FAURIE
David TINGAUD
Guy DIRRAS

The authors whose names are listed immediately below report the following details of affiliation or involvement in an organization or entity with a financial or non-financial interest in the subject matter or materials discussed in this manuscript. Please specify the nature of the conflict on a separate sheet of paper if the space below is inadequate.

Author names:

This statement is signed by all the authors to indicate agreement that the above information is true and correct (*a photocopy of this form may be used if there are more than 10 authors*):

Author's name (typed)	Author's signature	Date
Tarik SADAT	[signature]	02/02/2016
Azziz HOCINI	[signature]	04/02/2016
Lola LILENSTEN	[signature]	08/02/2016
Damien FAURIE	[signature]	02/02/2016
David TINGAUD	[signature]	03/02/2016
Guy DIRRAS	[signature]	02/02/2016
